# Dibenzo [a, c] phenazin-11-yl(phenyl) methanone (SBLJ23), a novel selective inhibitor targeting JAK2^V617F^ mutation in myeloproliferative neoplasms

**DOI:** 10.32604/or.2024.056256

**Published:** 2025-02-28

**Authors:** MOHAMMAD ABOHASSAN, MESFER MOHAMMAD AL SHAHRANI, SARAH KHALED ALOUDA, PRASANNA RAJAGOPALAN

**Affiliations:** Department of Clinical Laboratory Sciences, College of Applied Medical Sciences, King Khalid University, Abha, 61413, Saudi Arabia

**Keywords:** JAK2^V617F^ mutation, Myeloproliferative neoplasms, Kinase inhibitors, High-throughput virtual screening, Molecular dynamics simulation

## Abstract

**Background:**

The JAK2^V617F^ mutation plays a crucial part in the pathogenesis of myeloproliferative neoplasms (MPN), which includes polycythemia vera (PV), essential thrombocythemia (ET), and primary myelofibrosis (PMF) leading to aberrant proliferation and survival of hematopoietic cells. Alongside the challenges of drug resistance and side effects, identifying novel compounds that selectively target JAK2^V617F^ could provide more effective and safer therapeutic options for patients with MPNs.

**Materials and Methods:**

We employed computational approaches like high-throughput virtual screening, molecular dynamics simulations (MDS), and binding free energy calculations to identify inhibitors targeting wild and mutant JAK2 kinases. JAK2^V617F^ positive HEL, wild type JAK2 positive TF-1, and non-cancerous Vero cells were used for *in vitro* validations.

**Results:**

SBLJ23 emerged as a top candidate inhibitor with specificity for JAK2^V617F^. Protein-ligand interaction studies and MDS revealed stable interactions and binding of SBLJ23 over the simulation period, with Root Mean Square Deviation (RMSD) indicating consistent binding after 1t15ns. SBLJ23 displayed a half maximal inhibitory concentration (IC_50_) value of 522.4 nM against the JAK2 enzyme. The compound exhibited inhibition of cell proliferation in HEL and TF-1 cells, with half maximal cell growth inhibitory concentration (GI_50_) values of 2.51 and 15.87 µM, respectively. Moreover, SBLJ23 induced G_2_/M cell cycle arrest in HEL cells to facilitate apoptosis in these cell lines. The compound significantly reduced the percentage of phospho JAK2 and phospho STAT3 in HEL cells.

**Conclusion:**

High binding affinity, stable interaction profile, favorable binding free energy, and *in vitro* validations claim SBLJ23 as a potential lead compound against JAK2^V617F^ and suggest further development and optimization towards clinical application in managing myeloproliferative neoplasms.

## Introduction

The conditions collectively referred to as myeloproliferative neoplasms (MPN) include essential thrombocythemia (ET), primary myelofibrosis (PMF), and polycythemia vera (PV). The overproduction of platelet-forming cells and red blood cells in the bone marrow is a characteristic of these disorders [1]. These disorders are marked by a clonal expansion of hematopoietic cells and carry a heightened propensity for progressing to acute myeloid leukemia (AML). The pathogenesis of myeloproliferative neoplasms (MPN) involves several key-signaling pathways that drive the abnormal proliferation and survival of hematopoietic cells [2]. Central to these pathways is the Janus kinase-signal transducer and activator of transcription (JAK-STAT) pathway, which is often dysregulated due to mutations in genes such as JAK2, MPL, and CALR [3,4]. The JAK2, in particular, is prevalent across PV, ET, and PMF, leading to constitutive activation of the JAK-STAT signaling cascade. This activation promotes unchecked cell division and resistance to apoptosis [5]. Additionally, the mitogen-activated protein kinase (MAPK) and phosphoinositide 3-kinase (PI3K) pathways contribute to the malignant phenotype by enhancing cell proliferation, survival, and differentiation. Disruptions in these pathways result in the excessive production of erythrocytes, leukocytes, and platelets, characteristic of MPNs [6]. JAK2, part of the Janus kinase family comprising JAK1, JAK2, JAK3, and Tyk2, is crucial in transmitting signals after the erythropoietin, thrombopoietin, and other similar receptors are activated. These receptors are essential for the regulation and production of erythrocytes (red blood cells) and megakaryocytes (platelet-forming cells) [7]. When these receptors are activated, JAK2 transmits signals that promote the proliferation, differentiation, and survival of these hematopoietic cells, ensuring proper blood cell production and function [8].

Primary myelofibrosis (PMF), essential thrombocythemia (ET), and PV are MPN forms that do not have the BCR-ABL1 mutation. The most researched mutation in BCR-ABL1-negative neoplasms is JAK2V617F, which is believed to be present in approximately 95% of PV, 50% of ET, and 50% of PMF cases [9]. Patients with acute lymphoblastic leukemia, acute myeloid leukemia, and acute mega-karyoblastic leukemia also showed JAK2 mutations [10]. Additional research has shown that the JAK2-STAT pathway is crucial in inhibiting cell death and encouraging cell proliferation in different myeloid leukemias and solid tumors [11]. These findings underscore the significance of JAK2 mutations beyond MPN, highlighting their impact on a broader spectrum of hematologic malignancies. The aberrant activation of the JAK2-STAT pathway contributes to the malignant phenotype by enhancing cellular proliferation, survival, and resistance to programmed cell death, thereby facilitating the progression and maintenance of these cancers.

The presence of the V617F point mutation within the JH2 pseudokinase domain of JAK2 leads to continuous kinase activity, which promotes cell survival and proliferation without relying on cytokine binding [12]. This mutation causes JAK2 to remain perpetually active, leading to continuous signaling through the JAK-STAT pathway. As a result, hematopoietic cells proliferate uncontrollably and resist apoptosis, contributing to the pathogenesis of MPN. This aberrant activation promotes the excessive production of blood cells, characteristic of conditions such as thrombocythemia, polycythemia vera, and primary myelofibrosis [13].

The potential for treatment in MPN has been highlighted by the discovery of V617F, a mutation localized inside the pseudo-kinase domain of JAK2. Ruxolitinib, a potent JAK1/2 inhibitor, was the first Jakinib approved by the FDA for MPNs. Its effectiveness in treating these diseases has been well-established [14,15]. The efficacy of several first-generation Jak inhibitors, like tofacitinib, oclacitinib, and baricitinib, has been demonstrated. However, they inhibit multiple JAKs affecting a wide range of cytokines [16]. This lack of selectivity may contribute to their adverse effects. This has led to the suggestion of whether targeting a single JAK could offer better outcomes. Hence, the goal is to create improved next-generation Jak inhibitors with enhanced selectivity to maintain their therapeutic benefits and enhance their safety profile.

Therefore, in this work, using an interdisciplinary approach that integrates computational virtual screening, MDS, and binding free energy calculations, we aimed to identify novel inhibitors specifically targeting the JAK2^V617F^ mutation. By leveraging high-throughput screening techniques and advanced computational tools, we sought to discover compounds that exhibit high affinity and selectivity for the mutant JAK2 kinase, crucial for driving MPN and validating them for biological efficacy. This approach not only enhances our understanding of the structural dynamics and binding interactions involved in JAK2^V617F^ inhibition but also lays the foundation for developing potent and specific therapies that could potentially mitigate the pathogenic effects associated with this mutation.

## Materials and Methods

### Materials

The reagents, and buffers used in this study for the biochemical analysis were purchased from Sigma Aldrich (St. Louis, MO, USA) unless specified. The Vero (African green monkey kidney cell), HEL, and TF-1 (human erythroleukemia cell lines) were obtained from the American Type Culture Collection (ATCC, Rockville, MD, USA). Ruxolitinib, SET2 cells (human megakaryoblastic cell line), and UKE1 cells (hematopoietic cell line) were a kind gift from Prof. C. Harish, Centre for Stem Cell Research, College of Medicine, KKU, KSA. The JAK2 assay kit (#79520) was sourced from BPS Bioscience (San Diego, CA, USA). Annexin V assay kit (#CBA059) was supplied by Merck Millipore (Burlington, MA, USA). FITC-anti-phospho JAK2 antibody (Tyr 1007, Tyr 1008) (#44-426G) was acquired from Thermo Fisher Scientific (MA, USA), and PE anti-STAT3 Phospho (Tyr705) (#651003) antibody was purchased from BioLegend (San Diego, CA, USA).

### Methods

#### High-throughput virtual screening

SiBioLead LLP’s diversity-based high-throughput docking method (https://sibiolead.com/) (accessed on 26 September 2024) was used to screen ChemBridge compounds. Autodock-vina was employed as the docking algorithm for screening compounds. Initially, approximately 850,000 diverse molecules (scaffolds) from the ChemBridge library (https://chembridge.com/) (accessed on 26 September 2024) were docked against the target protein to predict docking energies. Subsequently, based on these energies, the top 10 scaffolds were selected. For each of these scaffolds, structurally related molecules were retrieved from the pre-built ChemBridge library using Tanimoto scores, and these molecules underwent a second round of docking against the target-protein. A docking box of 20 Å on both sides was used, and all docking computations were carried out in high-throughput mode with a vina exhaustiveness of 1. Docking energies were used to parse and score the stage II docking results.

#### Molecular dynamic simulations

The protein-ligand complex underwent Molecular Dynamics (MD) simulation using the GROMACS simulation package (V.2021) accessed through the MD simulation server at www.sibioled.com (accessed on 26 September 2024) [17]. The ligand topology was generated using AMBERTOOLS and ACPYPE (V. 2023.10.27), and system parameterization was achieved with the OPLS/AA force field. Initially, the protein-ligand complex was placed in a triclinic box filled with Simple Point Charge (SPC) water molecules to ensure proper hydration. The simulation system was enhanced with 0.15 M NaCl salt concentration and neutralized with NaCl counterions to mimic physiological circumstances. The entire system underwent energy minimization using the steepest descent method with 5000 steps. Subsequently, equilibration was performed for 300 ps under constant temperature (NVT) and pressure (NPT) conditions. The MD simulation ran for 100 ns using a leap-frog integrator, saving trajectory frames every 10 ps. Trajectory analysis utilized GROMACS (version 2021) built-in scripts, and graphical results were visualized using xmgrace (version 5.1.25).

#### Binding energy calculations

Using the GMX_MMPBSA technique, the protein-ligand complexes’ Gibbs binding free energy (ΔG binding) was ascertained [18]. To compute the ΔG binding values, this technique involved evaluating trajectory frames saved at 2 ns intervals during the 100 ns simulation period.

#### JAK2 kinase assay

The assay was carried out by the manufacturer’s instructions using a luminescence-based JAK2 assay kit (Catalog #79520) from BPS Bioscience, San Diego, CA, USA. In short, a 96-well plate was added with 25 µL of the master mix in distilled water, which contained PTK (poly Glu-4:1) substrate, 500 µM ATP, and kinase assay buffer with 1 M Dithiothreitol (DTT) Sigma Aldrich (St. Louis, MO, USA, #3483-12-3). Following that, 5 µL of SBLJ23 (Chembridge Corporation, San Diego, CA, USA) or the reference molecule (staurosporine, Sigma Aldrich (St. Louis, MO, USA, #62996-74-1) was added in log dilution at different doses ranging from 0.01 to 10,000 nM. The reaction was started by the addition of 2.5 ng/µL JAK2 enzyme and incubating at 30°C for 45 min. Following this, 50 µL of Kinase-Glo MAX (Promega, Madison, Wisconsin, MA, USA #V6071) was added to each well and incubated at room temperature for 15 min. Luminescence was recorded using a FLUOstar Omega plate reader (BMG Labtech, CA, USA). IC_50_ values were calculated using GraphPad Prism software (version 6.0, La Jolla, USA).

#### Cell proliferation assays

MPN and Vero cells were cultured in a complete growth medium under standard conditions, with assays performed when cells reached 80% confluency. Cell proliferation was measured using the MTT assay as previously described [19]. Vero, HEL, SET2, UKE1, or TF-1 cells (5 × 10^3^ cells/well) were seeded in 96-well tissue culture plates with regular growth medium (DMEM, Sigma Aldrich, St. Louis, MO, USA, #D5796) and treated with 0.001 µM to 100 µM in log dilutions of SBLJ23 for 48 h. Following medium removal, cells were incubated with 100 μL of MTT solution (1 mg/mL) for 4 h. The resulting formazan products were dissolved in 200 μL of DMSO (Sigma Aldrich, St. Louis, MO, USA, #D8418), and absorbance was measured using, FLUOstar Omega model plate reader, BMG LABTECH, at 560 nM. GraphPad Prism 6.0, was used to calculate the GI_50_ values.

#### Cell cycle assay

The assay was done using a kit (Elabscience, Hudson, TX, USA, #E-CK-A351) according to the manufacturer’s instructions. HEL (0.5 × 10^6^ cells/well) were added to a 6-well plate and incubated for 24 h. The cells were then treated with various concentrations of SBLJ23 and incubated for 24 h. After incubation, the cells were washed twice with 1 × sterile PBS, treated with 50 μL of cell cycle assay reagent, and incubated in the dark for 15 min. The cells were then washed twice with wash buffer and resuspended in HBSS buffer (Sigma Aldrich, St. Louis, MO, USA, #H8264). Data acquisition was performed on a flow cytometer (Guava easyCyte™ HT System, Merk Millipore, MA, USA), capturing ten thousand events per sample. The data were analyzed using ExpressPro Software from Millipore (Burlington, MA, USA), and the percentage of the cell population in different cell cycle phases was determined.

#### Apoptosis analysis by annexin V assay

Apoptosis was performed using a kit (#CBA059 Merck Millipore, Burlington, MA, USA) according to the manufacturer’s instructions. HEL cells (0.5 × 10^6^) were seeded in 6-well plates and treated with SBLJ23, followed by incubation in 5% CO_2_ at 37°C for 24, 48, or 72 h. After incubation, the cells were harvested, washed with kit buffer (#CBA059 Merck Millipore, Burlington, MA, USA), and incubated with 0.25 µg/mL Annexin V reagent for 15 min in the dark. The cells were resuspended in a kit buffer containing 0.5 µg/mL propidium iodide after two washes with 1x sterile PBS. Ten thousand events were acquired on a flow cytometer (Guava easyCyte™ HT System, Merk Millipore, MA, USA), and data analysis was performed using InCyte software (v.2.7, Merk Millipore, MA, USA).

#### Phospho JAK2/phospho STAT3 inhibition assay by flow cytometry

HEL cells were treated with 1.25 and 2.5 µM of SBLJ23 along with controls and incubated for 24 h in a 5% CO_2_ incubator at 37°C, with untreated controls included for comparison.

Following incubation, each cell was resuspended in HBSS buffer and twice rinsed with sterile 1 × PBS. The cells were then treated with 2 μg/mL anti-phospho JAK2-FITC or 1 μg/mL anti-phospho STAT3-PE antibodies and incubated in the dark for 30 min. The cells were then resuspended in HBSS buffer after being washed twice with 1x PBS. A flow cytometer (Guava easyCyteTM HT System, Merk Millipore, MA, USA) was used to collect 10,000 events, and ExpressPro Software (v.2.7, Merk Millipore, MA, USA) was used to analyze the data. The proportion of positive cells in each quadrant was displayed.

#### Statistical analysis

Statistical analyses were performed using GraphPad Prism 6.0 (La Jolla, USA). Results are expressed as mean ± standard deviation (SD). Data were analyzed using ANOVA followed by multiple comparisons. Statistical significance was set at *p* ≤ 0.05.

## Results

### Structural analysis of JAK2^V617F^ complexed with known inhibitor shows “hot-spot” residues 

**Figure 1:**
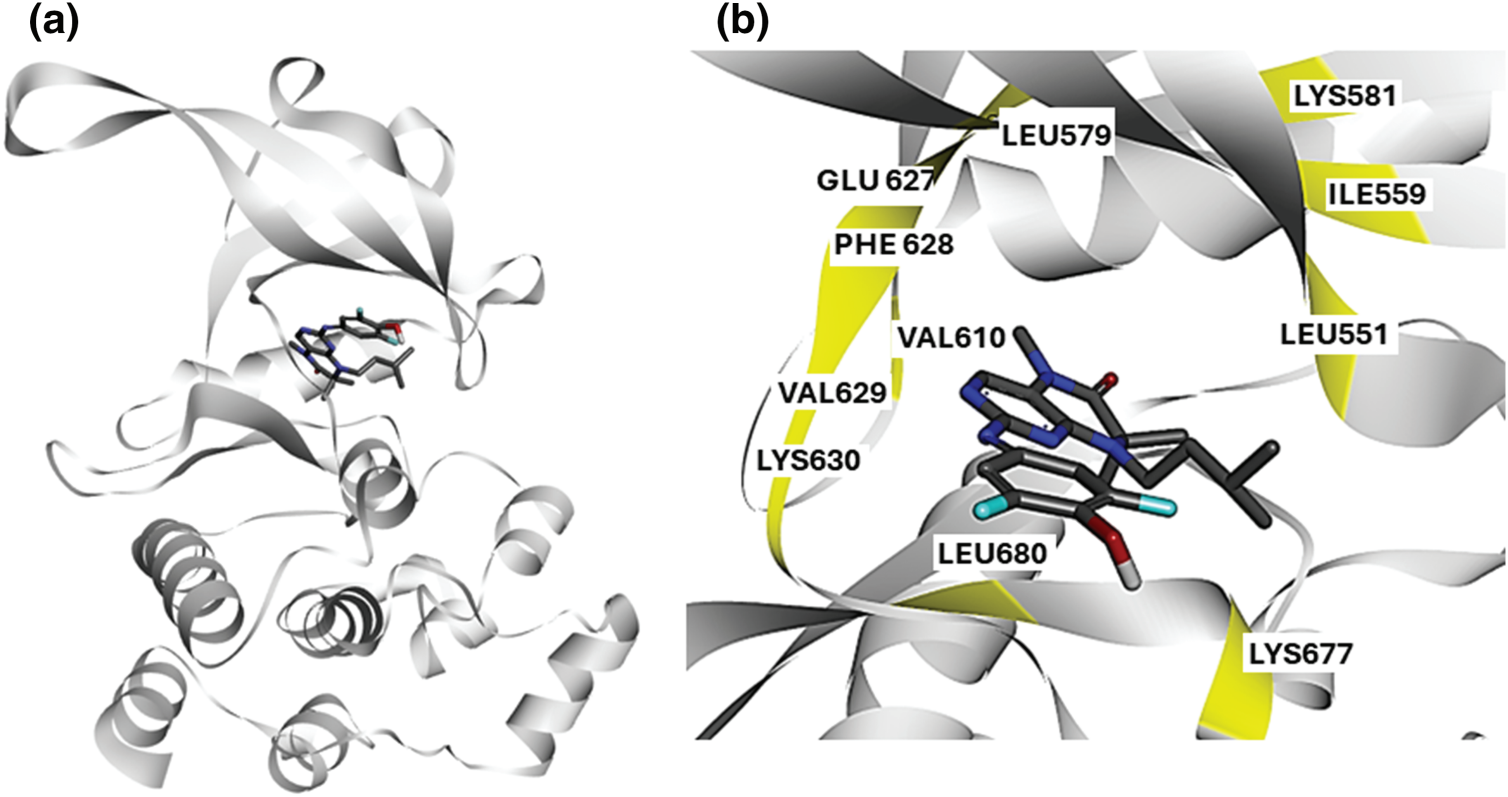
Structural Analysis of JAK2^V617F^ Inhibitor Interactions: (a) Crystal structure of JAK2^V617F^ bound to EKT retrieved from the experimental structure 6G3C. The kinase domain of JAK2 is shown in blue, with the inhibitor EKT highlighted in green. (b) Protein-ligand interaction profile of compound 2 with JAK2^V617F^. Key interacting residues (Lys630, Val629, Phe628, Glu627, Ile559, Leu551, Lys677, and Leu680) are highlighted, illustrating the critical binding interactions within the kinase domain.

To identify novel small molecules that bind to JAK2^V617F^ and inhibit its kinase activity, we analyzed the interaction profile of a known JAK2^V617F^ inhibitor, as retrieved from the experimental structure 6G3C (Fig. 1a).

We obtained the crystal structure of JAK2^V617F^ bound to EKT Protein-ligand interaction profiling revealed that the known inhibitor, EKT, interacts with critical residues in the kinase domain of JAK2 kinase (Fig. 1b). Our analysis indicates that compound 2 engages with Lys630, Val629, Phe628, Glu627, Ile559, Leu551, Lys677, and Leu680 (Fig. 1b). Based on these findings, we hypothesize that small molecules targeting the kinase domain of JAK2^V617F^, with a similar interaction profile to EKT, could serve as potential JAK2 inhibitors specific for the V617F mutation.

### High-throughput virtual screening (HTVS) predicts small molecules with high affinity for JAK2^V617F^ kinase

**Figure 2:**
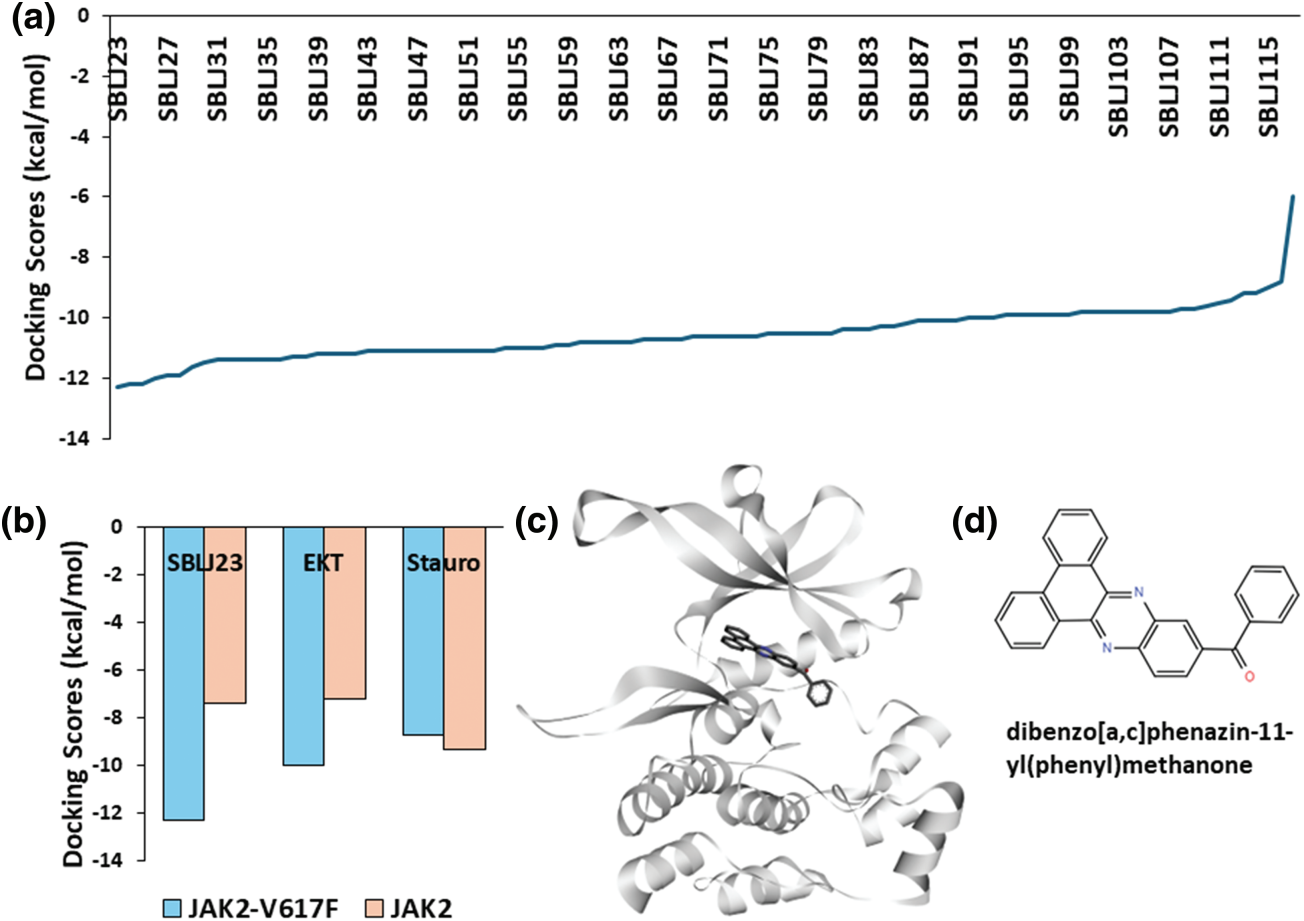
High-Throughput Virtual Screening Results: (a) Docking scores of the top molecules from the ChemBridge library screened against JAK2^V617F^ kinase. SBLJ23 is identified as the molecule with the highest binding affinity. (b) Comparative docking scores of SBLJ23, EKT, and staurosporine with JAK2^V617F^ and wild-type JAK2. SBLJ23 shows superior binding affinity to JAK2^V617F^ while maintaining selectivity over wild-type JAK2. (c) Binding mode of SBLJ23 within the kinase domain of JAK2^V617F^. The compound fits well within the active site, forming stable interactions. (d) 2D representation of SBLJ23, illustrating the molecular structure and key functional groups.

To identify chemical compounds with high affinity for the JAK2 kinase V617F mutation, we conducted HTVS using the ChemBridge library, targeting the kinase domain of JAK2. Utilizing the diversity-based HTVS technique (D-HTVS) available at www.sibioled.com (accessed on 26 September 2024), we screened ChemBridge compounds with molecular weights ranging from 350 to 750 kDa. The docking box was configured to cover the entire ATP binding site, and docking calculations were performed using the Autodock-vina algorithm. The results were ranked based on docking scores, and the top molecules are shown in Fig. 2a.

Our screening results revealed that Dibenzo [a, c] phenazin-11-yl(phenyl) methanone (Internal code SBLJ23) exhibited the highest affinity for JAK2^V617F^ among the molecules screened. Consequently, SBLJ23 was selected for further biological and cell-based analysis (Fig. 2a). Given that the retrieved target JAK2^V617F^ structure includes a known inhibitor (EKT), we compared the binding scores of our SBLJ23 with those of standard known inhibitors of JAK2^V617F^. We performed docking calculations with EKT and the standard JAK2 inhibitor staurosporine using the same parameters.

Additionally, we investigated whether our compound binds to the JAK2 wild type, as we are targeting the JAK2^V617F^ mutation. Our results demonstrated that SBLJ23 has superior binding affinity for JAK2^V617F^ compared to EKT or staurosporine when compared to wild-type JAK2 (Fig. 2b). Analysis of the binding mode revealed that SBLJ23 fits well within the kinase domain of the JAK2^V617F^ structure (Fig. 2c). The 2D representation of SBLJ23 is shown in Fig. 2d.

### Protein-ligand interaction profiling (PLIP) of SBLJ23 

**Figure 3:**
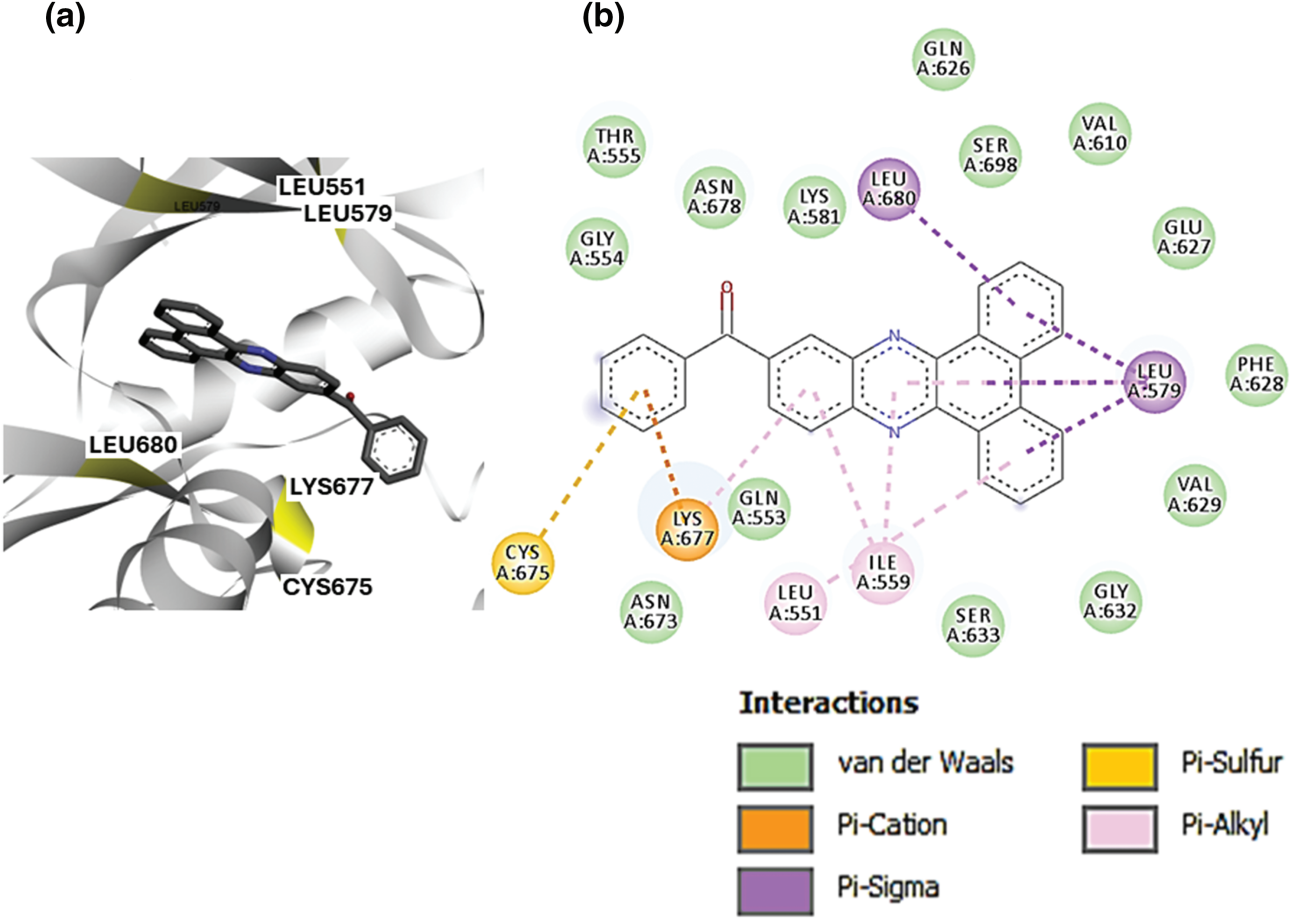
PLIP of SBLJ23: (a) Detailed interaction profile of SBLJ23 with JAK2^V617F^ using Discovery Studio Visualizer. Key residues Cys675, Lys677, Leu680, and Leu579 are highlighted. (b) Types of interactions formed by SBLJ23 with JAK2^V617F^, including pi-sulfur, pi-cation, and pi-alkyl bonds, contribute to the compound’s binding stability.

Based on the preferential docking scores, we selected SBLJ23. PLIP using Discovery Studio Visualizer revealed that SBLJ23 interacts with key residues of the JAK2 kinase V617F mutation (Fig. 3a). Specifically, SBLJ23 engages with residues such as Cys675, Lys677, Leu680, and Leu579. The interaction profile indicates that SBLJ23 forms pi-sulfur and pi-cation bonds, along with several pi-alkyl bonds, contributing to its strong binding affinity (Fig. 3b). To further understand the potential of SBLJ23 as a lead molecule, we analyzed the stability and specificity of these interactions. The pi-sulfur and pi-cation bonds formed with Cys675 and Lys677, respectively, suggest strong and specific binding within the kinase domain. The pi-alkyl bonds with Leu680 and Leu579 further stabilize the compound within the active site.

### MDS predict the stable binding of SBLJ23 to JAK2^V617F^

**Figure 4:**
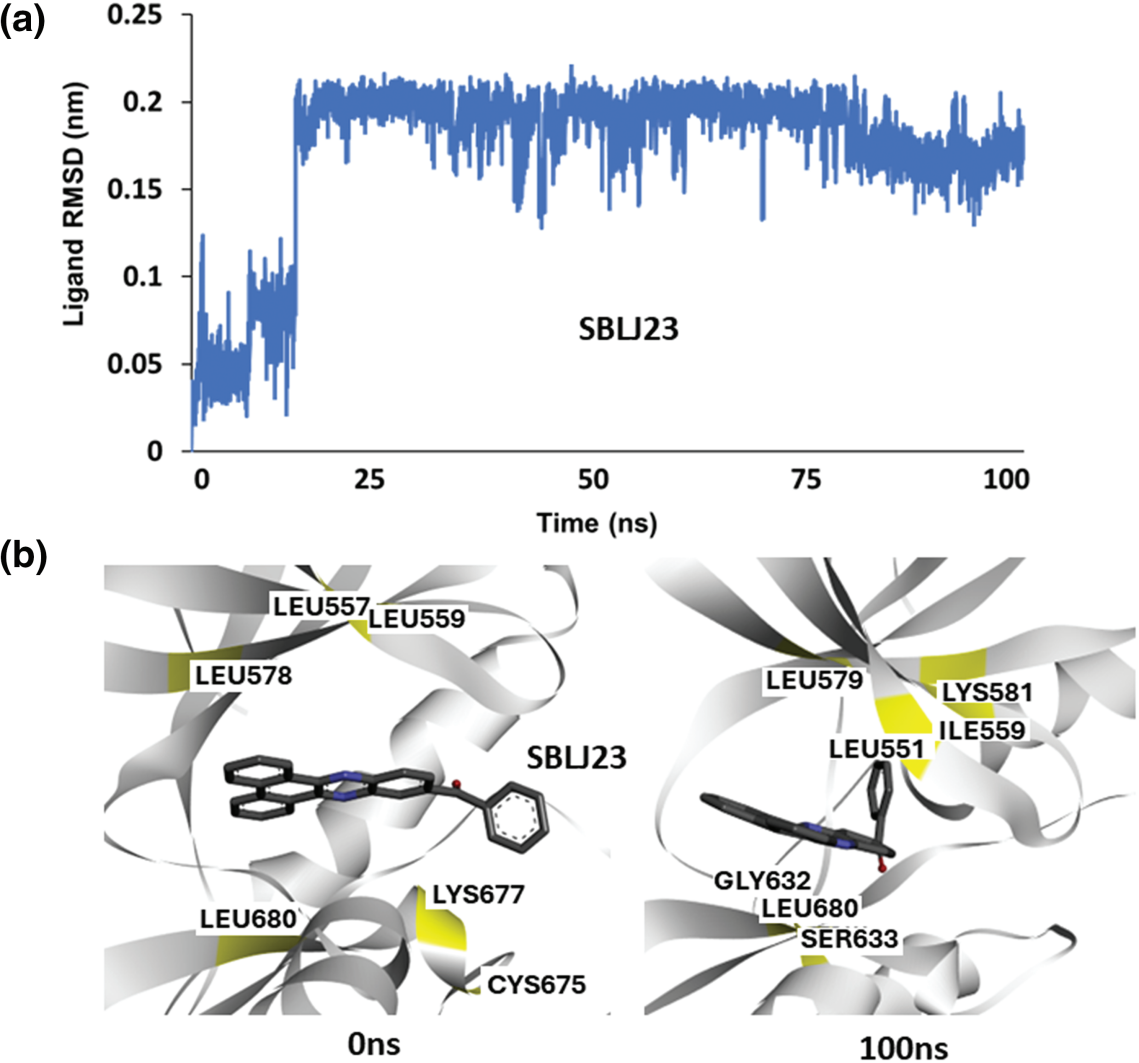
Molecular Dynamics Simulation of SBLJ23 Binding: (a) Root Mean Square Deviation (RMSD) of SBLJ23 bound to JAK2^V617F^ over 100 ns. SBLJ23 shows stable binding after 15 ns, which stabilizes after 50 ns. (b) Snapshots of trajectory frames before and after the 100 ns MD simulation. The 0 ns frame shows the initial binding pose of SBLJ23, while the 100 ns frame shows additional interactions and a more stable binding conformation.

To understand the binding stability of the predicted lead molecule SBLJ23 to JAK2^V617F^ kinase, we conducted a 100 ns atomistic MDS of the JAK2^V617F^. Simple point charge (SPC) water and NaCl counterions were used in a triclinic box for the simulations. The simulation system was supplemented with 0.15 M NaCl to preserve physiological conditions. Using the automated web application for MD simulations, wSiBioLEAD web server, the MD simulation was run for 100 ns. Simulation trajectories were analyzed using GROMACS’ in-built trajectory analysis tools. The Root Mean Square Deviation (RMSD) of the ligand relative to the protein indicated stable binding of SBLJ23 to JAK2^V617F^ after 15 ns, suggesting that SBLJ23 comfortably fits within the kinase domain (Fig. 4a). This data demonstrates that SBLJ23 stabilizes more rapidly and consistently within the kinase domain. Additionally, snapshots of trajectory frames before and after the simulation were analyzed. Results revealed that SBLJ23 adopts a more stable binding pose as the simulation progresses. Comparing the 0 and 100 ns trajectory frames, additional interactions between SBLJ23 and JAK2^V617F^ kinase were observed (Fig. 4b).

### Solvent-based binding energy calculation shows SBLJ23 has favorable binding to JAK2^V617F^ kinase

**Figure 5:**
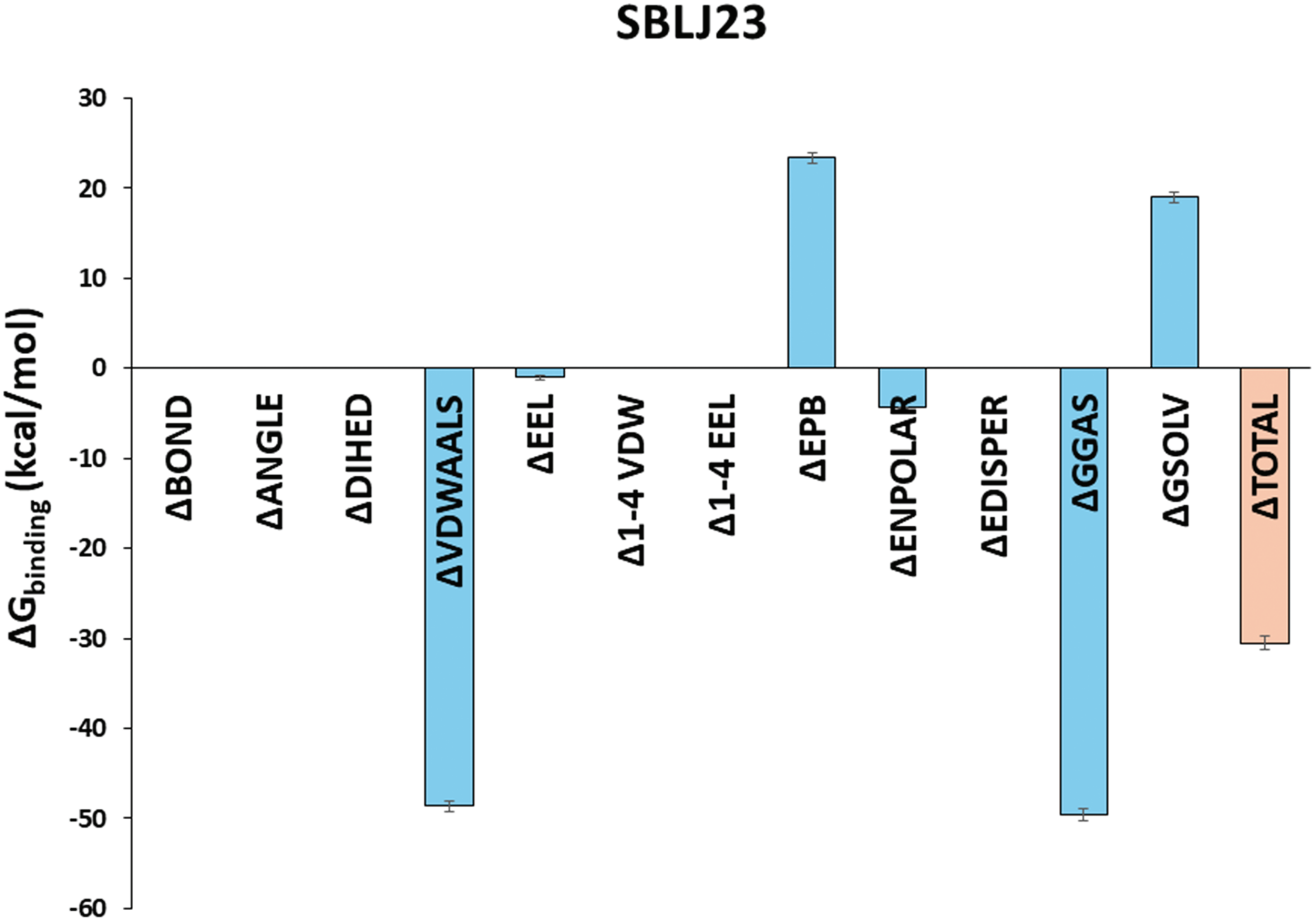
Binding Free Energy Calculations of SBLJ23. The binding free energy of SBLJ23 was calculated using the MM-PBSA approach. The predicted binding energy of −30.55 kJ/mol indicates a favorable and strong binding interaction.

To calculate the binding free energies of SBLJ23 under solvated conditions, we utilized the 100 nanosecond trajectory frames from our MD simulations. The Molecular Mechanics Poisson-Boltzmann Surface Area (MM-PBSA) approach, integrated into the SiBioLEAD MD simulation tool, was employed for this purpose. Our analysis predicted a binding energy of −30.55 kJ/mol for SBLJ23, indicating a highly favorable binding affinity (Fig. 5).

### SBLJ23 effectively inhibited cell-free JAK2 kinase enzyme and controlled cell proliferation in MPN cells

**Figure 6:**
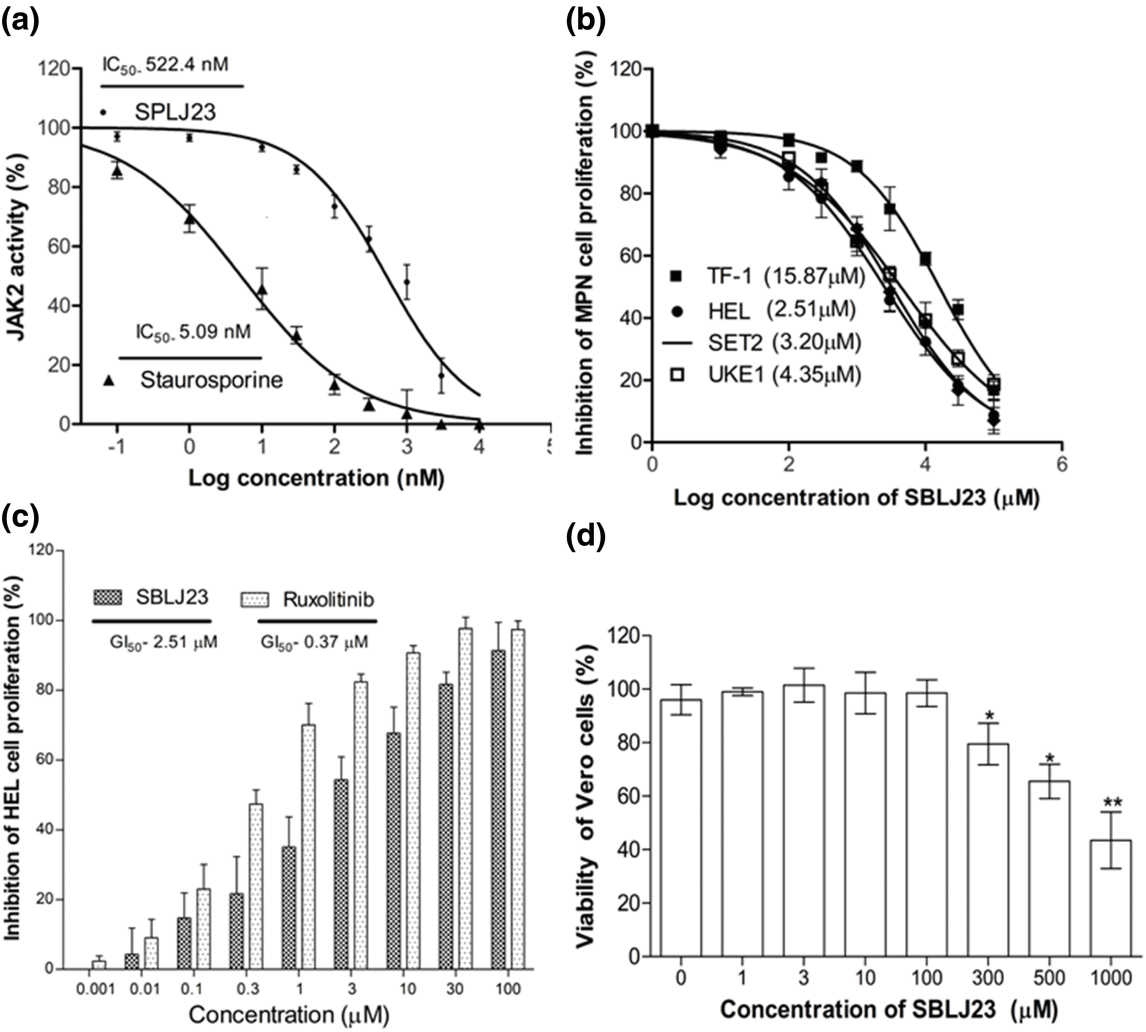
Biological efficacy of SBLJ23: (a) The enzyme IC_50_ values of SBLJ23 and staurosporine against the JAK2 enzyme, representing the mean ± standard deviation (SD) from three independent experiments. IC_50_ values were determined using GraphPad Prism version 6.0 software. (b) The GI_50_ values for cell proliferation in HEL, SET2, and UKE1 cells with the JAK2^V617F^ mutation and TF-1 cells with wild-type JAK2 when treated with SBLJ23. (c) Comparative inhibition of HEL cell proliferation when treated with different concentrations of SBLJ23 and Ruxolitinib. (d) The effect of SBLJ23 on the viability of Vero cells at various concentrations. Cell proliferation and viability assays were assessed using the MTT assay, with mean ± SD values analyzed using GraphPad Prism version 6.0 software. *p < 0.05, **p < 0.01.

SBLJ23 effectively inhibited JAK2 activity in a dose-dependent manner, with an IC_50_ value of 522.4 nM (Fig. 6a), while the standard compound staurosporine showed an IC_50_ value of 5.09 nM (Fig. 6a). The efficacy of SBLJ23 against both wild-type (TF-1) and JAK2^V617F^ mutant (HEL) (JAK2 mutated AML), SET2 and UKE1 cell proliferation was analyzed using the MTT assay. SBLJ23 dose-dependently inhibited HEL. SET2 and UKE1 cell proliferation with GI50 values of 2.51, 3.21 and 4.35 µM, respectively (Fig. 6b), and TF-1 cell proliferation with a GI_50_ value of 15.87 µM (Fig. 6b) dose-dependent inhibition of HEL cell proliferation was observed with SBLJ23 and the widely used JAK2 inhibitor Ruxolitinib, with GI_50_ values of 2.51 µM and 0.37 µM, respectively (Fig. 6c). To determine if these biologically active doses were tolerated by normal, non-cancerous cells, various doses of SBLJ23 were tested on Vero cell proliferation. The compound showed no effect on Vero cell proliferation up to 100 µM (Fig. 6d).

### SBLJ23 arrested the cell cycle at the G_2_/M phase and promoted apoptosis in HEL cells

**Figure 7:**
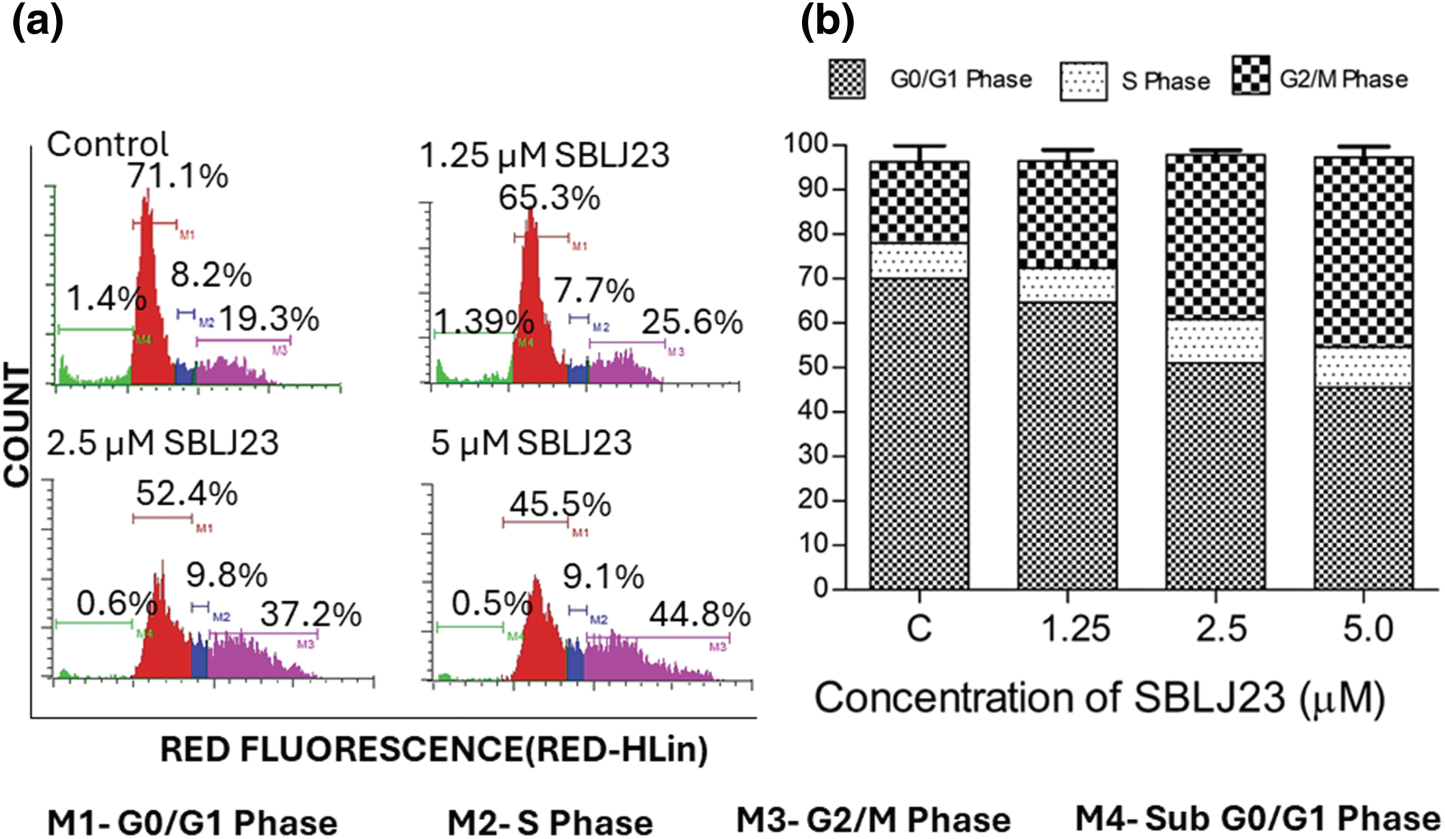
(a) Histograms from flow cytometry assay displaying the cell cycle distribution of HEL cells treated with different concentrations of SBLJ23 for 24 h. Numerical values indicate the mean ± standard deviation (SD) percentage of cells in various cell cycle phases. (b) Percentage distribution of cell cycle phases in HEL cells treated with SBLJ23. Each experiment was performed in triplicate, with results expressed as mean ± SD.

Since maximum anti-proliferative activity was observed in the HEL cells, it was chosen for further cell-based validations. To investigate if the anti-proliferative efficacy of SBLJ23 impacted other cellular functions of HEL cells, a cell cycle analysis was performed. Treatment of HEL cells with the SBLJ23 resulted in a dose-dependent increase in the sub-G_2_/M population compared to the control (Fig. 7a,b).

**Figure 8:**
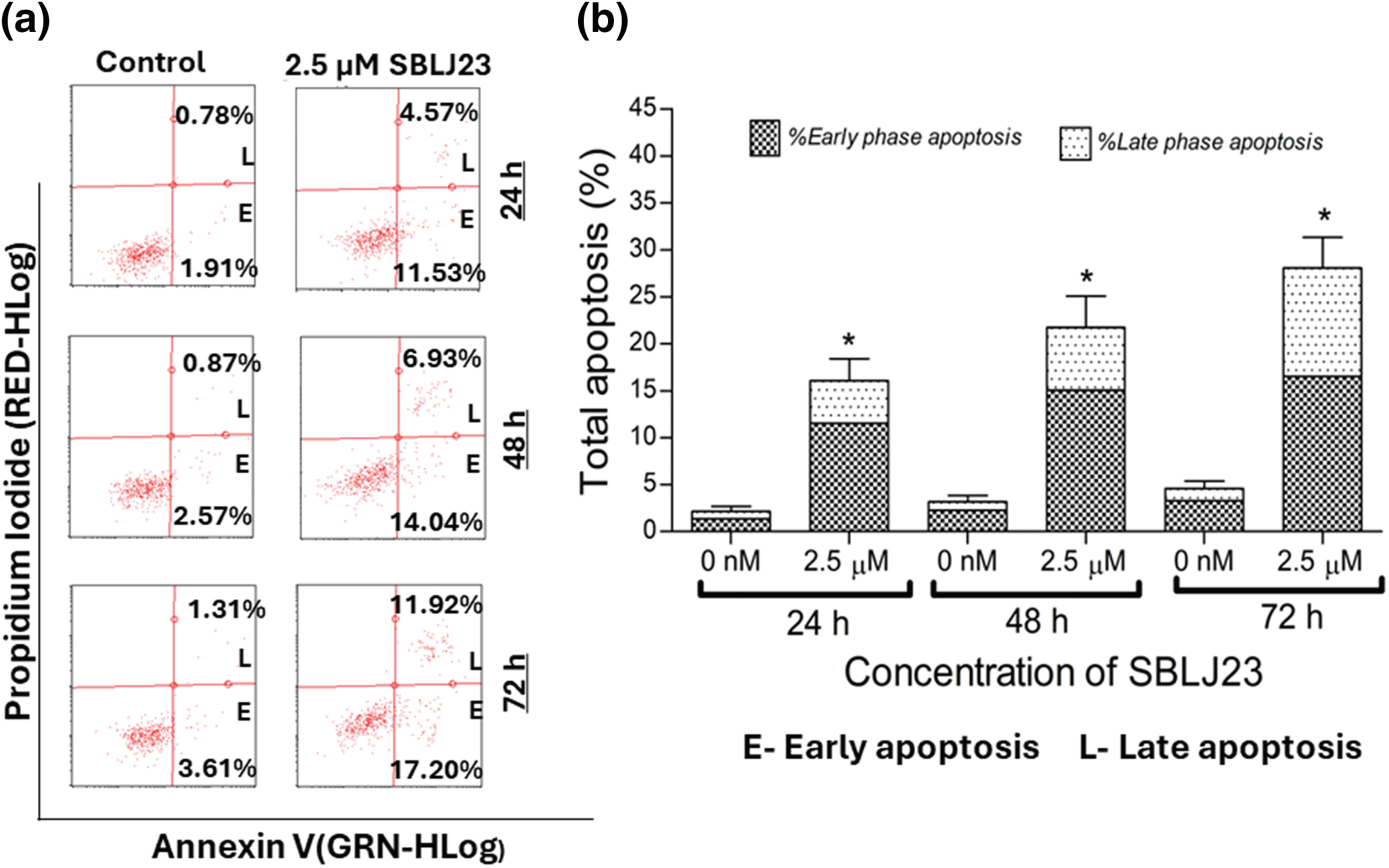
(a) Representative plots from the Annexin V assay showing apoptotic HEL cells after treatment with SBLJ23 at various time points. The compound induced a time-dependent increase in both early and late apoptotic populations. (b) Bar chart illustrating the proportions of early and late apoptotic cells within the total apoptotic population at different time points following SBLJ23 treatment. Each experiment was performed in triplicate, with results expressed as mean ± SD. *p < 0.05.

Additionally, when analyzed for time-dependent apoptosis, SBLJ23 treatment increased the percentage of early and late apoptotic HEL cells in a time-dependent manner (Fig. 8a,b).

### Target inhibition by SBLJ23 in HEL cells

Target inhibition assays were performed using flow cytometry. GI_50_ value and GI_25_ value of SBLJ23 in HEL cells were used as reference concentrations to check out the dose efficacy.

**Figure 9:**
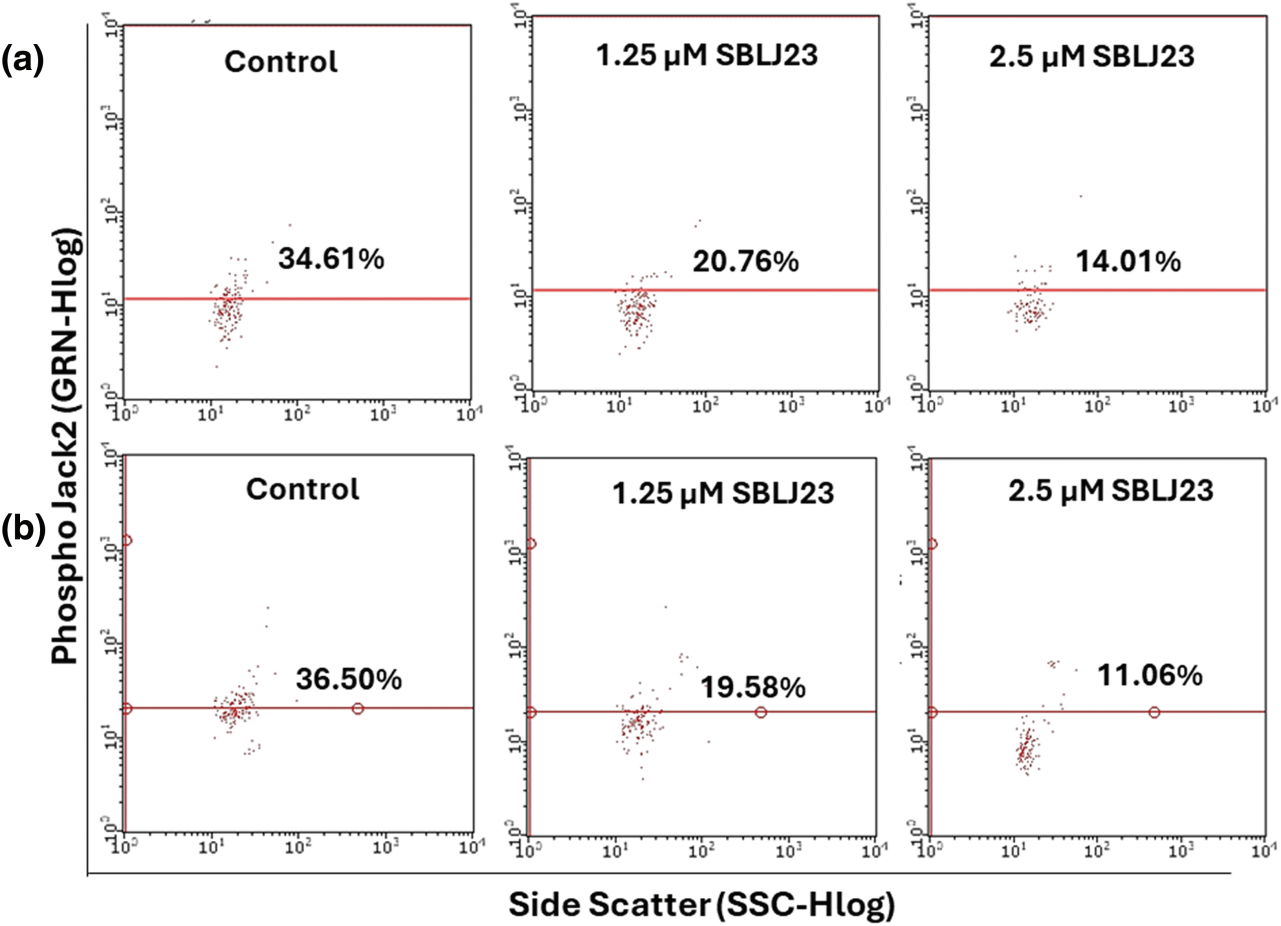
The percentage of (a) phospho JAK2 or (b) phospho STAT3-positive population in HEL cells treated with SBLJ23 was assessed using flow cytometry. Numerical values represent the mean ± standard deviation (SD) from multiple experiments. Pretreatment with different doses of SBLJ23 led to a reduction in both phospho JAK2 and phospho STAT3 populations in HEL cells.

The control HEL cells had 34.61% positive population for phospho JAK2, while treatment of 1.25 µM SBLJ23 decreased the phospho JAK2 population to 20.76% (Fig. 9a). Treatment with 2.5 µM SBLJ23 further decreased the phospho JAK2 positive cells to 14.01% in HEL cells (Fig. 8a). Similarly, treatment of 1.25 and 2.5 µM of SBLJ23 to HEL cells resulted in 19.58% and 11.06% phospho STAT3 positive population respectively, while the untreated control HEL cells had 36.50% phospho STAT3 positive population (Fig. 9b).

## Discussion

The JAK2^V617F^ mutation leads to constitutive activation of the JAK-STAT pathway, promoting uncontrolled cell proliferation. Therefore, targeting the JAK2^V617F^ mutation with specific inhibitors presents a promising therapeutic strategy. Our study identifies and characterizes SBLJ23 as a potential novel inhibitor with a strong binding affinity and stable interaction profile against JAK2^V617F^, based on comprehensive MDS and binding free energy calculations.

Our initial high-throughput virtual screening of the ChemBridge library, targeting the kinase domain of JAK2, identified SBLJ23 as the top candidate. Utilizing diversity-based high-throughput virtual screening (D-HTVS), we screened compounds with molecular weights between 350 to 750 kDa. The docking results, performed using the Autodock-vina algorithm, ranked SBLJ23 highest based on docking scores. Comparative docking studies with known inhibitors such as EKT and staurosporine confirmed that SBLJ23 exhibits superior binding affinity to JAK2^V617F^ while also showing significant selectivity over the wild-type JAK2 [20]. This initial screening highlighted the potential of SBLJ23 as a specific inhibitor for the JAK2^V617F^ mutation, meriting further investigation.

PLIP results revealed that SBLJ23 forms stable interactions with critical residues within the kinase domain of JAK2^V617F^, such as Cys675, Lys677, Leu680, and Leu579. These interactions included pi-sulfur and pi-cation bonds, as well as multiple pi-alkyl bonds, contributing to the strong binding affinity [21]. The detailed interaction analysis provided insights into the structural stability and specificity of SBLJ23, indicating its potential as a lead compound for targeting the JAK2^V617F^ mutation. These interactions are crucial for the inhibitory activity of SBLJ23, as they stabilize the compound within the active site of the kinase, thereby effectively inhibiting its function [22,23]. Moreover, this specificity is critical for developing targeted therapies with potentially lower cytotoxicity compared to broader JAK2 inhibitors [24].

To further validate the binding stability of SBLJ23, we performed a 100 ns MD simulation of the JAK2^V617F^ complex under physiological conditions. The Root Mean Square Deviation (RMSD) analysis showed that SBLJ23 maintains a stable binding conformation within the kinase domain after 15 ns. This rapid stabilization indicates that SBLJ23 not only fits well within the kinase domain but also remains firmly bound throughout the simulation [25,26]. Additional interaction analysis from the MD trajectory frames revealed that SBLJ23 adopts a more favorable binding pose over time, forming additional interactions with the JAK2^V617F^ kinase, thereby enhancing its inhibitory potential, which indicates a stable and favorable binding configuration [27]. This enhanced interaction profile over time underscores the potential of SBLJ23 as a robust inhibitor targeting the JAK2^V617F^ mutation [20].

The binding free energy calculations using the MM-PBSA approach further corroborated our findings, predicting a highly favorable binding energy of −30.55 kJ/mol for SBLJ23. This value reflects the strong and stable interactions between SBLJ23 and the JAK2^V617F^ kinase observed during the MD simulations [20]. The combination of high docking scores, detailed interaction profiling, stable binding during MD simulations, and favorable binding free energy collectively highlights the potential of SBLJ23 as a potent and specific inhibitor of the JAK2^V617F^ mutation. These computational predictions were complemented with *in vitro* findings, where SBLJ23 inhibited JAK2 kinase in the cell-free enzyme assay. It’s interesting to note that after treatment with SBLJ23, the proliferation of JAK2^V617F^ positive HEL, SET2, and UKE1 cells was dramatically suppressed in a concentration-dependent manner. TF-1 cell proliferation was also suppressed by SBLJ23, however at a greater dose range. The multi-fold increase in the GI_50_ value for the antiproliferative effect of SBLJ23 against the TF-1 cells harboring wild-type JAK2 can be attributed to the selectivity of the compound towards JAK2^V617F^ inhibition [28]. Myoproliferative diseases are primarily caused by JAK2V617F mutations, and in cells carrying these mutations, suppression of JAK2 activity results in growth inhibition and apoptosis [29]. Inhibition of the AK2^V617F^ MPN cell proliferation by SBLJ23 was in line with the aforementioned report, with the highest activity observed in the HEL cells. We therefore proceeded with HEL cells for further evaluations. Ruxolitinib (RUX) and Fedratinib are used to treat patients with MPN. However, approximately half of them stop taking it due to either ineffectiveness or intolerance [30]. The effect of SBLJ23 in HEL growth inhibition was evaluated with the widely used JAK2 inhibitor ruxolitinib. The efficacy of ruxolitinib in controlling HEL cell growth corroborated the available literature, thereby validating the efficacy of SBLJ23 in HEL growth inhibition [31]. Alongside, the cell viability assay indicated no toxicity in the non-cancerous Vero cells up to 100 µM of SBLJ23 treatment, which indicates a safe therapeutic window for the biological efficacy of the compound in MPN cells [32]. A dose-dependent increase in SBLJ23 treatment for 24 h indicated G_2_/M phase cell cycle arrest of the HEL cells, with a decrease in the G_0_/G_1_ phase of HEL cells. The compound also increased early and late-phase apoptosis in HEL cells time-dependently. Many studies show that compounds can arrest cells in the G_2_/M phase before inducing apoptosis [33,34]. As the cell cycle is a major checkpoint to deciding life and death, it was concluded that SBLJ23 favors G_2_/M cell cycle arrest to promote apoptosis in these cells [35].

Next, we investigated whether SBLJ23’s JAK2-inhibitory effects might also affect the JAK2-STAT signaling pathway. We examined the phosphorylation state of downstream proteins and JAKs in HEL cells in order to comprehend this. According to our findings, JAK2 and STAT3 protein phosphorylation was markedly reduced by SBLJ23. Inhibition of STAT3 as a downstream modulator has been proven to evaluate the clinical potential of JAK2 inhibitors [36]. The effect of SBLJ23 on the primary cells derived from JAK2^V617F^ mutated MPN patients and elucidation of SBLJ23 on the pathways, which are dysregulated in myeloproliferative neoplasms, was not investigated in this study, which stands as a limitation of this work. However, the promising results from this preliminary investigation, warrant further preclinical studies and optimization efforts to develop SBLJ23 as a therapeutic agent for managing MPN.

## Conclusion

In conclusion, our study identifies SBLJ23 as a promising novel inhibitor of the JAK2^V617F^ mutation, which is a significant driver in MPN. Detailed protein-ligand interaction profiling revealed stable and critical interactions within the kinase domain of JAK2^V617F^, supported by 100 ns MDS that confirmed the compound’s stable binding conformation. The computational predictions were complemented by the *in vitro* investigations where SBLJ23 effectively inhibited JAK2 in cell-free and cell-based experiments along with apoptotic induction by cell cycle arrest in MPN cells. Collectively, these findings suggest that SBLJ23 has significant potential as a lead molecule for the development of targeted therapies against MPNs, offering a promising avenue for therapeutic intervention with potentially lower cytotoxicity compared to broader JAK2 inhibitors. However, further preclinical studies and optimization are warranted to advance SBLJ23 towards clinical application.

## Data Availability

Data used in this study is available with the communicating author upon reasonable request for non-commercial purposes.
